# A practical guide to assessing functional motor weakness: a review of validated techniques

**DOI:** 10.1007/s00415-025-13139-4

**Published:** 2025-05-27

**Authors:** James Dolbow, Soheil El-Azzouni, Yiyi Zhang, Christopher Geiger

**Affiliations:** 1 Department of Epilepsy, University Hospitals-Cleveland Medical Center, Case Western Reserve University, Cleveland, OH 44106 USA; 2 Department of Neuromuscular Neurology, University Hospitals-Cleveland Medical Center, Case Western Reserve University, Cleveland, OH 44106 USA; 3 University Hospitals-Cleveland Medical Center, Case Western Reserve University, Cleveland, OH 44106 USA

**Keywords:** Functional weakness, Physical Exam, Functional Neurological Symptom Disorder

## Abstract

Functional neurological symptom disorder, specifically, functional limb weakness, is a commonly seen condition in clinical neurological practice and requires careful examination for diagnosis. Specific and detailed examination techniques have been developed and validated over the past 100 years to help clinicians differentiate functional limb weakness from objective neurological weakness. These techniques vary in sensitivity, specificity, clinical application, and limitations. However, over time, the studied and/or validated forms of these exam techniques may have been lost and often not performed or taught in the way it was originally studied, thus decreasing the reliability of the examiner’s findings. With many new examination techniques for functional limb weakness having been studied in recent years, it is important to not only review the form of each of these examination techniques, but also discuss the clinical applications, limitations, and utility of each. To date, there has been no comprehensive review demonstrating the exact form of all the studied and/or validated examination techniques for functional limb weakness and their proposed clinical utility. This review analyzes 9 examination techniques for functional limb weakness that have been studied for validity and provides readers with the exact technique of examination used to study each. It also outlines their sensitivity, specificity, clinical applications, and limitations to help clinicians accurately diagnose functional limb weakness.

## Introduction

Functional Neurological Symptom Disorders (FNSD) are reported to comprise up to 15% of neurology clinic referrals and 9% of all neurology admissions [1–4], many of which present repeatedly to hospital emergency rooms seeking care [5]. Patients with FNSD often present with acute sensorimotor symptoms that are difficult to differentiate from acute stroke or other neurological emergencies. These presentations often require substantial emergency department and acute hospital resources, resulting in significant patient, healthcare, and societal cost [5–8]. In recent years, many studies have reported on the lack of attention that healthcare, specifically the fields of neurology and psychiatry, have historically paid to the diagnosis and treatment of FNSD [9–11].

With the recent recognition and validation of several clinical examination techniques utilized to differentiate FNSD from objective neurological motor, sensory and movement disorders, and the subsequent identification of “positive signs” of motor weakness, FNSD is no longer considered a diagnosis of exclusion [12–16]. The utilization of these techniques, and the “positive signs” of FNSD they uncover, are clinically important as the 5th edition of the *Diagnostic and Statistical Manual of Mental Disorders* (DSM-5) no longer requires the identification of an acute psychological stressor to make the diagnosis, and intentional symptom falsification is present in only a small minority of cases of FNSD [17]. Although there are several views on the most accurate and sensitive terminology to use when describing the features FNSD, no consensus currently exists [18, 19]. Thus, for the purposes of this review, weakness without a structural or electrophysiological cause will be referred to as functional limb weakness (FLW), and weakness with a structural or electrophysiological cause will be referred to as objective neurological weakness (ONW).

### Aim of review

Although studies and reviews have discussed the sensitivity, specificity, and positive predictive value of many examination techniques used to distinguish FLW and ONW [13, 20, 21], the exact validated techniques of examination and the interpretation of their results have not been discussed beyond their original publications. The aim of this review is to discuss the exact form of each validated examination technique and the practical application of all previously validated examination techniques of FLW (see Table 1).

**Table 1:**
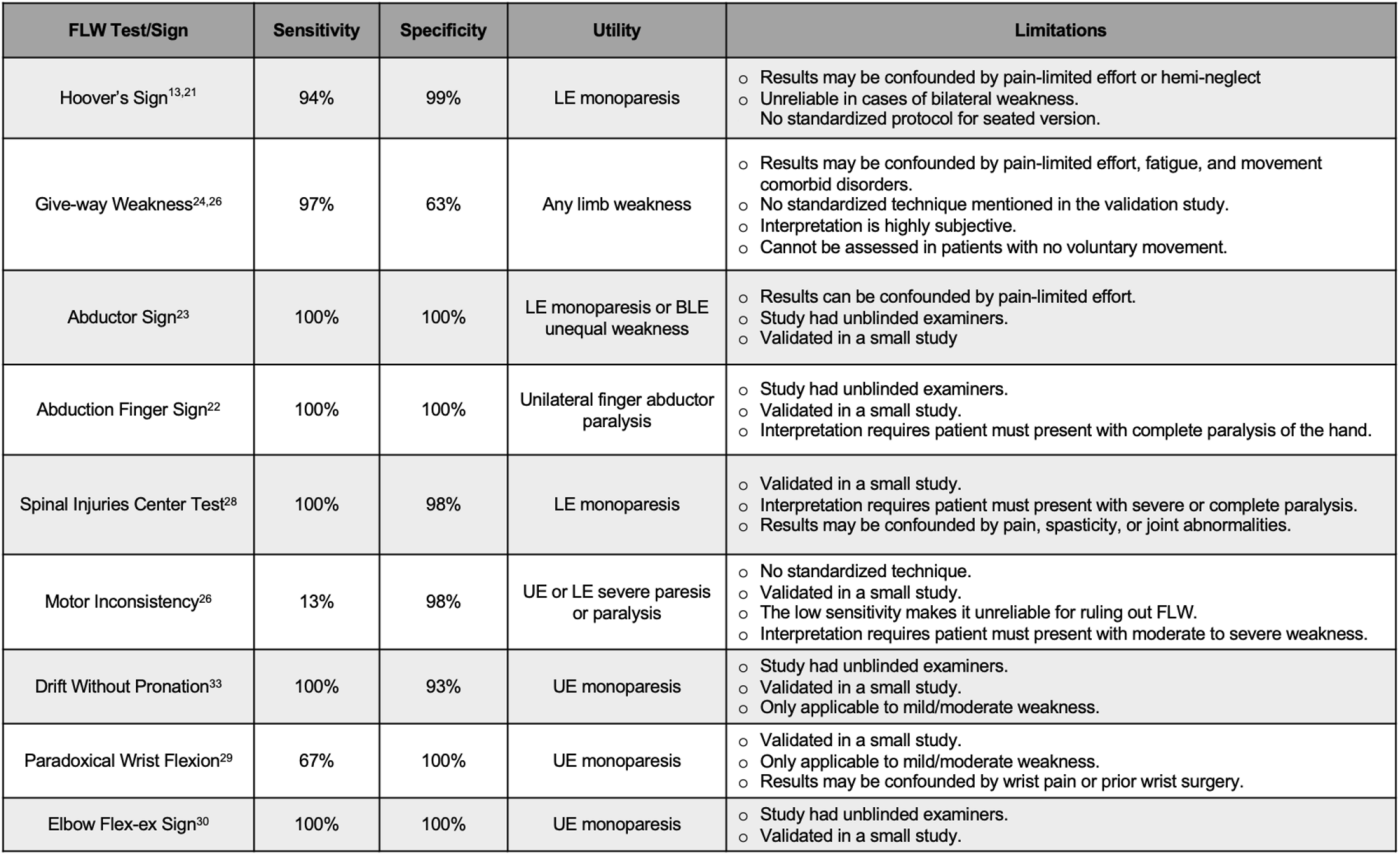
Validated Techniques for Assessing Functional Limb Weakness

## Methods

The Cochrane Library, Pubmed, and Google Scholar databases were searched from inception to March 2024 for articles related to FNSD. Our searched terms included “inorganic”, “psychogenic”, “functional”, “hysterical” “hysteria” and “conversion”. The resulting studies were reviewed and included if they provided sufficient information on the technique of examination for reproducibility and if a validation analysis was performed on any technique used to distinguish FLW from ONW. Studies without adequately detailed description of the examination technique to reliably reproduce the technique, or those without a validation analysis, were excluded. Case reports, abstracts, and non-English translation studies were also excluded. Ultimately, 13 studies reporting on 9 examination techniques met the inclusion criteria [22–34]. Although 6 studies did not provide a detailed description of the exact examination technique used [25, 27, 31, 32, 35, 36], 4 of these included examination descriptions considered sufficient for inclusion [25, 27, 31, 32].

## Examining functional motor weakness

### Hoover’s sign

First described by Charles Franklin Hoover in 1908 and originally termed *Hoover’s phenomenon*, Dr. Hoover noted that in a supine patient, the force exerted downward by one lower extremity (LE) into the bed is directly related to the force exerted upward by the contralateral LE. However, he observed that when patients with LE FLW were asked to lift their affected LE, no downward force was felt by his hand held under the patient’s contralateral Achilles tendon [37, 38].

*Hoover’s sign* is the most extensively studied and validated examination technique for FLW [22]. Of the 5 studies identified, only 1 prospectively evaluated Hoover’s sign [22], while 2 primarily focused on other techniques [23, 24], and 1 broadly examined the characteristics of patients with FNSD [25]. The mechanism of Hoover’s sign was also validated by computerized quantitative analysis using tools measuring isometric force showing significant differences between FLW from ONW [26].

### Technique

**Fig. 1:**
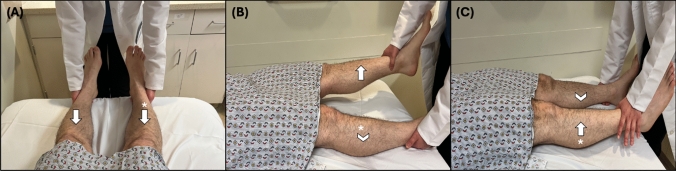
**A** Baseline assessment: Patient is asked to extend each LE into the bed, one at a time. **B** Test 1: Patient is asked to lift their unaffected leg while specific attention is paid by the examiner to the extension force of the affected leg (*), and comparing this to the results from (**A**). **C** Test 2: Patient is asked to lift the affected leg (*) while specific attention is paid by the examiner to the extension force of the unaffected leg. Here the arrow signifies the action and direction of the instructed action, and the arrowhead signifies the action and direction being assessed

As originally described by Dr. Hoover and analyzed for its validity, the originally validated examination technique, known as Hoover’s sign involves a baseline strength assessment and two sequential tests (Fig. 1). Though many texts have described the assessment of Hoover’s sign as only one of the two tests required for completion, no study has compared the sensitivity or specificity of using only one or both tests in the assessment of FLW [26, 39]. Additionally, the principles of Hoover’s sign have been adapted by many clinicians into a test completed while sitting, though there are currently no studies assessing the reliability of Hoover’s sign performed seated.

#### Baseline strength assessment

As the patient lies supine with knees extended and the examiner positions themselves at the foot of the bed, the examiner places their hands under both heels or ankles of the patient. Then, the examiner instructs the patient to extend each LE into the bed with full force, while noting the force applied to each palm as the patient extends their LE. This test is used to establish a “baseline” comparison of LE extension strength, which is compared to the results from test 1 and 2.

##### Test 1

With the examiner’s hands remaining under both the patient’s heels/ankles, the examiner then instructs the patient to lift their unaffected LE off the bed (flexing at the hip). Specific attention was paid to the force exerted by the contralateral (unlifted/affected) LE into the examiner’s palm, comparing it with the previous baseline strength assessment.

##### Test 2

With the examiner’s hands remaining under both the patient’s heels/ankles, the examiner instructs the patient to lift their affected LE off the bed (flexing at the hip). Specific attention paid to the force exerted by the contralateral (unlifted/unaffected) LE into the examiner’s palm, comparing it with the previous baseline strength assessment.

### Interpretation and limitations

Test 1 is considered positive if the downward force applied by the unlifted affected LE while lifting the unaffected LE is stronger than during the baseline assessment.

Test 2 is considered positive if the downward pressure applied by the unlifted unaffected is absent or minimal while attempting to lift the affected LE, and less than that found during the baseline assessment.

A Hoover’s sign is present if either test 1 or 2 is positive and thus indicative of FLW.

Hoover’s sign is the most widely studied examination technique used to assess for LE FLW. Though the grouped estimate of the sensitivity and specificity of Hoover’s sign has been reported to be 94% and 99%, respectively, only 1 study evaluated it prospectively (8 participants), reporting 63% and 100%, respectively [14, 22]. Because of the variability in sensitivity, this sign should not be interpreted in isolation, but rather in the context of other FLW exam findings. Additionally, the sensitivity of the 2-step Hoover’s sign was first interpreted in only 4 patients with FLW, and prospectively validated in only 8 patients [22, 37]. Given this overall low number of prospectively studied patients, evidence of the sensitivity and specificity of this exam technique is limited.

Hoover’s sign is a positive diagnostic feature of LE FLW, particularly when hip and hamstring weakness coexist. Also, Hoover’s sign should only be interpreted in patients with LE monoparesis, as a strong contralateral LE is needed to serve as a control limb. Pain-limited weakness or the presence of hemi-neglect can confound results.

## Give-way weakness/collapsing weakness

The evaluation of give-way weakness is predicated on known neurophysiological properties of muscle power and the inconsistencies of a patient’s strength exam with these known properties. In cases of FLW, the examined limb can exhibit abrupt fluctuations in force when resistance is applied. In contrast, ONW will demonstrate sustained resistance without abrupt changes in force.

### Technique

Unfortunately, no studies examining give-way weakness have explained the exact technique in which it was or should be tested, however, each study does describe at least one method of testing [25, 27, 28]. These studies describe an examination during which the patient maintains an isometric contraction (a contraction of variable force without a change in muscle length) of the examined upper or lower limb, while the examiner applies progressively more resistance to a specific, isolated muscle group. In patients demonstrating give-way weakness, there is a sudden cessation of isometric contraction, rather than a gradual failure to oppose resistance by the examiner. Given the constant properties of gravity, limbs held up isometrically against gravity may be the most accurate position for evaluation.

For example, when examining unilateral LE weakness, the examiner should first have the patient lie supine and lift their affected LE 1–2 feet off the bed. Positioned at the side of the bed nearest the affected LE, the examiner places their hand on the lifted LE and slowly provides progressively greater resistance downward against the lifted LE.

### Interpretation and limitations

The test is considered positive if, at any point during the application of a reasonable amount of limb resistance, the patient’s limb exhibits a complete cessation of resistance, such as abrupt falling of the limb to the bed. Give-way weakness is not observed if the limb constantly opposes resistance throughout its range of motion though it may gradually fail to resist the examiner’s force. Additionally, give-way weakness can be exhibited in patients that appear to provide full strength during examination, but as the affected limb meets progressively more resistance, it fails to oppose resistance in exhibits a rachet-like manner, exhibiting significant variation in limb strength.

Because give-way weakness is poorly described in terms of proper technique though it report to have a high pooled sensitivity of 97% and a relatively low pooled specificity of only 63%, exam finding should be interpreted with caution as a means of “ruling in” FLW [25, 27]. Additionally, given the exact technique these validation studies used is unclear, it is impossible to attribute their validating results to our clinical findings on exam.

To avoid false positive results, findings should be interpreted in the context of limb potential contributing factors including limb pain, superimposed movement disorder, such as tremor and myoclonus, and the patient’s limited attention or altered mentation. For example, a patient with significant shoulder pain may quickly drop their supinated and extended arm if resistance is applied downward if the pain of the applied resistance if too great. Conversely, in patients with neuromuscular disorders or fatigue, without shoulder pain, failure to overcome resistance should be gradual, not abrupt. Additionally, patients with complete paralysis cannot be assessed for give-way weakness.

Like Hoover’s sign, give-way weakness is interpreted subjectively by the examiner. However, further electrophysiological analysis by Van der Ploeg et al., utilized dynamometer data to evaluate the difference in limb resistance if limb strength was assessed using isometric resistance (the “make test”) compared to progressive resistance until the limb strength was overcome (the “break test”) [28]. This study demonstrated that patients with FLW displaying give-way weakness exhibited a “break-index” significantly different from patients with ONW, meaning those with FLW provided significantly more strength with break-testing than make-testing compared to those with ONW. This study also reported that the exhibition of give-way weakness had a sensitivity of 100% and specificity of 89%, thereby validating the subjective assessments made by examiners [25]. The limitation of the test of give-way weakness stems from a lack of data regarding the positive predictive values of this examination.

## Abductor sign

Similar to the Hoover’s sign, the abductor sign was validated in 2004 and is based on the principle of complementary opposition, the natural production of a contralateral synergistic movement in the body [24].

### Technique

**Fig. 2:**
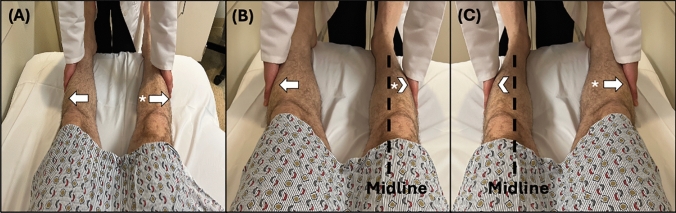
**A** Baseline assessment: Patient is asked to simultaneously abduct both LEs with full force. **B** Test 1: With the affected LE in the midline, the patient is instructed to abduct the unaffected leg while the examiner pays specific attention to the abductive force of the affected LE (*),and comparing it that found during (**A**). **C** Test 2: With the unaffected LE in the midline, the patient is instructed to abduct the affected leg (*) while the examiner pays specific attention to the abductive force of the unaffected LE, and comparing it to that found in (**A**). Here the arrow signifies the action and direction of the instructed action, and the arrowhead signifies the action and direction being assessed

With the patient lying supine and the examiner positioned at the foot of the bed, the examiner places their hands on the bilateral lateral calves of the patient. Complete examination of the abductor sign requires a baseline assessment followed by two tests, each focusing on the strength of abduction in a specific LE (Fig. 2). For reliable assessment, the patient’s LEs should be uncovered and the bed free of anything that could limit the patient’s range of motion.

#### Baseline assessment

While lying supine and both LEs extended, equidistant from the midline, and approximately 6–12 inches apart at the ankle, the patient is instructed to simultaneously abduct both LEs with full effort. The examiner provides an equal bilateral adductive force to both LE simultaneously and notes the patient’s strength of LE abduction.

##### Test 1

With the patient remaining supine, the examiner places the patient’s affected LE in the midline and the unaffected LE in an abducted position, approximately 6–12 inches apart at the ankles. The examiner then directs the patient’s attention solely to the unaffected (abducted) LE. The examiner then instructs the patient to abduct the unaffected LE while paying specific attention to the affected LE, noting any differences in the force of abduction compared to baseline assessment.

##### Test 2

With the patient still supine, the examiner switches the position of the patient’s LEs, placing the unaffected LE in the midline and the affected LE in the abducted position, approximately 6–12 inches apart at the ankles. The examiner then directs the patient’s attention solely to the affected (abducted) LE. The examiner then instructs the patient to abduct the affected LE while paying specific attention to the midline unaffected LE, noting any differences in the force of abduction when compared to the baseline assessment.

### Interpretation and limitations

In the baseline assessment, both patients with FLW and ONW should exert less oppositional abductive force on the examiner’s hand in the affected LE compared to the unaffected LE, or no abductive force at all.

Test 1 is considered positive if the affected (midline) LE exerts more abductive force on the examiner’s hand than when performed during the prior baseline assessment.

Test 2 is considered positive if the unaffected (midline) LE exerts less abductive force on the examiner’s hand than when performed during the prior baseline assessment.

This sign was validated in only one study of 33 participants (16 FLW and 17 ONW) with either weakness of one LE or bilateral unequal weakness. The study reported both 100% sensitivity and 100% specificity in detecting FLW [24]. However, it's important to note that the study was conducted with unblinded examiners, and no data were collected on inter-rater reliability. The reliability of the abduction sign can be confounded by pain or difficulty following commands or keeping attention.

## Abduction finger sign

Based on the principle of cross-body synkinesis, the abduction finger sign was validated by Tinazzi et al*.* in 2008 [23]. Unlike Hoover’s sign, which is based on the principle of complementary opposition [37, 38], the abduction finger sign relies on the principle of involuntary contralateral synkinetic movements [40].

### Technique

**Fig. 3:**
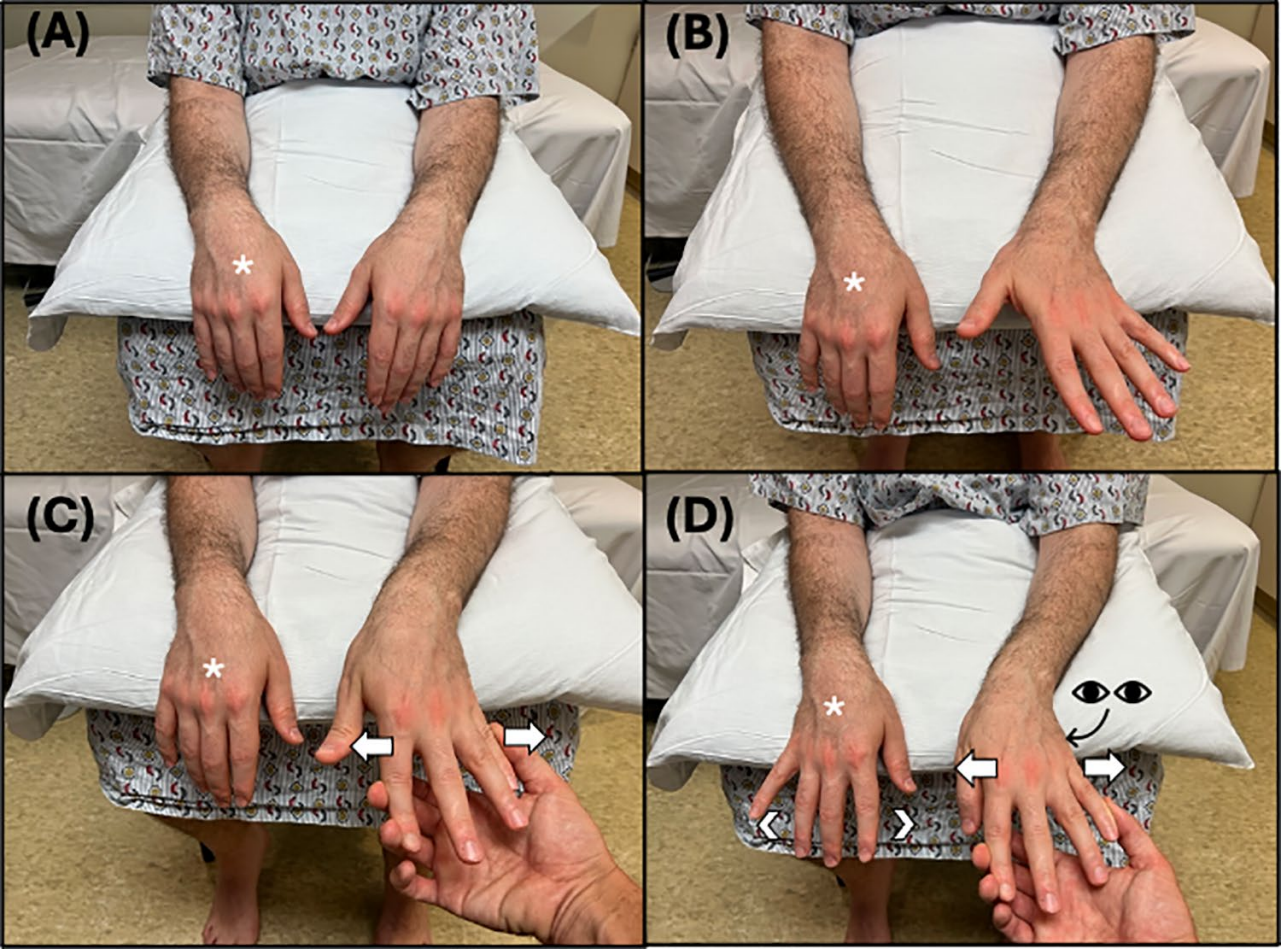
**A** Patient positioning. **B** Baseline Assessment: Patient is instructed to abduct the fingers on both the affected (*) and unaffected hand. **C** Shows the examiner providing resistance to finger abduction of the unaffected hand, with no abduction seen in the affected hand (*), representing a negative test. **D** Shows abduction of the affected fingers (*) with resisted abduction of the unaffected limb, representing a positive test. Here, the arrow signifies the action and direct of the instructed action, and the arrowhead signifies the action and direction being assessed

The seated patient is positioned with both UEs resting in front of them, while their forearms and/or wrists are placed on a surface, such as their knees or a board, with palms facing down (pronated) and hands/fingers suspended in the air over the distal edge of the supporting surface (Fig. 3).

#### Baseline assessment

The examiner instructs the patient to spread their fingers (abduct their fingers) on both hands with full effort. Note is taken of any movement observed in the paralyzed hand, especially abduction of the 5th digit.

##### Test

The examiner positions their fingers on the outsides of both the 2nd and 5th digits of the unaffected, leaving the affected hand resting palm down. Directing the patient’s attention to the unaffected hand, the examiner instructs the patient to spread (abduct) their fingers on the unaffected hand with maximum force against the opposing adductive force of the examiner for 2 min. While providing adductive force to the unaffected fingers, the examiner pays specific attention to the affected hand, observing for a synkinetic abduction of the fingers, especially that of the fifth digit.

### Interpretation and limitations

The abduction finger sign is considered positive if synkinetic movement is observed in the affected limb during resisted finger abduction of the unaffected limb. Synkinetic movements are expected in those with FLW and those without weakness. The absence of synkinetic movement may indicate ONW unrelated to FLW.

The blinded validation study comprised 57 participants, including 10 with FLW, 11 with severe ONW, and 36 healthy controls. The study reported that the abduction finger sign was present in all participants from both the healthy control group and the FLW group, while absent in all participants with ONW, demonstrating 100% sensitivity and specificity. Though the researcher performing the examination was not blinded to the etiology of the participants’ weakness, the interpretation of sign positivity was conducted by a blinded researcher present during the examination [23].

Interestingly, Hoover’s sign was present among the study participants with hemibody FLW and absent in individuals with ONW [18]. Additionally, 5 patients with FLW and 5 patients with ONW were further tested with surface electromyography (EMG) of the first dorsal interosseous and abductor digiti minimi during examination. Surface EMG analysis confirmed the presence of muscle activity in patients with FLW, however, those with ONW had no response.

Since synkinetic movements can occur in cases of incomplete limb paralysis, this sign can only be accurately interpreted in patients who exhibit complete paralysis.

## Spinal injuries center test

The spinal injuries center test, validated by Yugue et al. in 2004, aids in distinguishing FLW from LE ONW by utilizing a patient’s natural tendency to balance tone against gravity [31].

### Technique

This technique is informally based on the natural reflex of a patient to maintain a partially supported limb in the position where it is placed by the examiner.

#### Baseline assessment

**Fig. 4:**
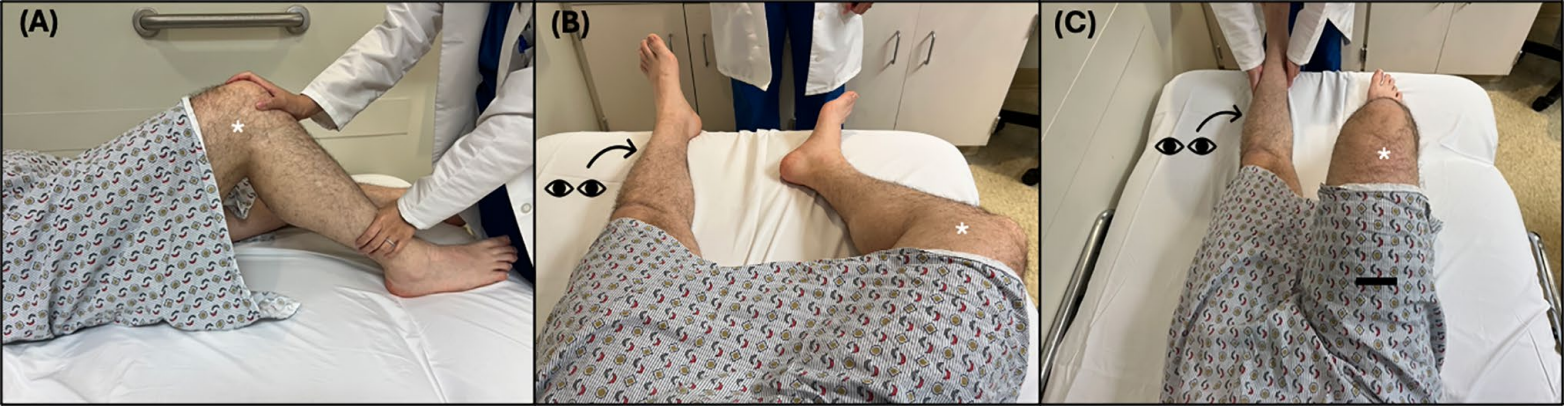
Initial position: **A** The examiner positions the affected LE (*) so that the knee is flexed, foot is flat on the bed, and the knee is pointing upward. Testing: **B** show a situation in which after drawing the patient’s attention to the unaffected LE, the affected LE (*) slides down the bed and falls laterally, representing a negative test. **C** Shows a situation in which after drawing the patient’s attention to the unaffected LE, the affected LE (*) remains upright against gravity, representing a positive test if strength is disproportional to that found on initial assessment

With the patient lying supine with LEs extended, and the examiner positioned at the foot or side of the bed, the patient is asked to lift each LE off the bed without any resistance provided by the examiner (Fig. 4). It is important to note that for this examination to be valid, the patient must exhibit severe weakness, and thus inability to lift their LE off the bed.

##### Test

The examiner positions the affected LE with the knee flexed, keeping the patient's foot flat on the bed and the knee pointed upward, holding it in place. Directing the patient’s attention to the unaffected LE, the examiner removes their hand from the affected LE.

### Interpretation and limitations

The spinal injuries center test is considered positive if after the support of the examiner is removed, the patient is able to hold the affected LE in place without it immediately falling to the bed, despite being initially unable to lift the affected LE off the bed during baseline assessment.

Spinal Injuries Center sign is a highly sensitive (100%) and specific (98%) indicator of FLW, though only validated by 1 study with 14 participants with FLW [29]. Because this sign does not use the principle of complementary opposition, this may make it particularly useful in patients who have negative or equivocal Hoover’s sign or abductor sign.

Patients with mild to moderate weakness may still be able to keep their knee flexed in an upright position when passively placed there, yet they may be unable to lift the entire LE against gravity. Therefore, this test should only be considered valid in patients with severe weakness or complete paralysis of the LE.

## Motor inconsistency

Of all the validated studies, the evaluation of motor inconsistency is the least specifically described in both technique and interpretation. Chabrol et al. studied the presence of multiple positive signs of FLW in both patients with FLW and ONW and described the finding of motor inconsistency as the “impossibility to do a certain movement while another movement using the same muscle is possible” [27].

### Technique

The technique used to establish motor inconsistency lacks an explicit description in its validation study. However, it is understood that its overall principle suggests FLW is often specific to a particular movement rather than to a specific nerve or muscle. To highlight this principle, the authors of this review propose the techniques below. It is important to note that these techniques are based solely on the principle of motor inconsistency and may not precisely mirror the techniques utilized in the validation study.

#### Baseline assessment

In patients with severe weakness or paralysis, the full assessment of UE and LE strength through manual muscle testing should be completed with the patient positioned supine.

#### Strength consistency evaluation

With the examiner positioned to ensure the patient's safety during movement, the patient is instructed to transition to a seated position at the edge of the bed, while the examiner closely observes any deviations in motor movements compared to the baseline assessment. In patients presenting with LE weakness, special attention should be given to the patient's ability to perform hip adduction, abduction, flexion, or extension while shifting to the bedside. In patients presenting with UE weakness, special attention is given to the patient's capability to lift or support themselves on the affected limb during this transition. Additionally, with safety precautions ensured, the patient may be encouraged to stand and walk under supervision, carefully noting any inconsistency with the baseline assessment.

### Interpretation and limitations

Motor inconsistency is present when a patient, who initially cannot perform a specific task, such as moving a limb against gravity or using a particular muscle group during the baseline assessment, demonstrates these abilities when transitioning from supine to sitting at the edge of the bed, standing, or walking.

The validation study for motor inconsistency, like give-way weakness, does not provide a detailed description of the standardized exam used. This makes both the examination and the interpretation of motor inconsistency difficult to attribute clinically. Furthermore, given motor inconsistency was only tested in 7 study participants with FLW and reported a sensitivity of only 13%, this sign likely has a low reliability of ruling out FLW, despite its 98% specificity.

This test may be best used to assess patients with severe weakness, as compensatory maneuvers by complementary muscle groups may be present to help the patient overcome mild weakness when completing complex movements.

## Drift without pronation sign

Pronator drift is a well-known sign of neurological upper motor neuron weakness [41]. Babinski first described drift without pronation in 1907, proposing it as a sign of FLW [42]. This sign was later validated in 2013 by Daum and Aybek [34]

### Technique

**Fig. 5:**
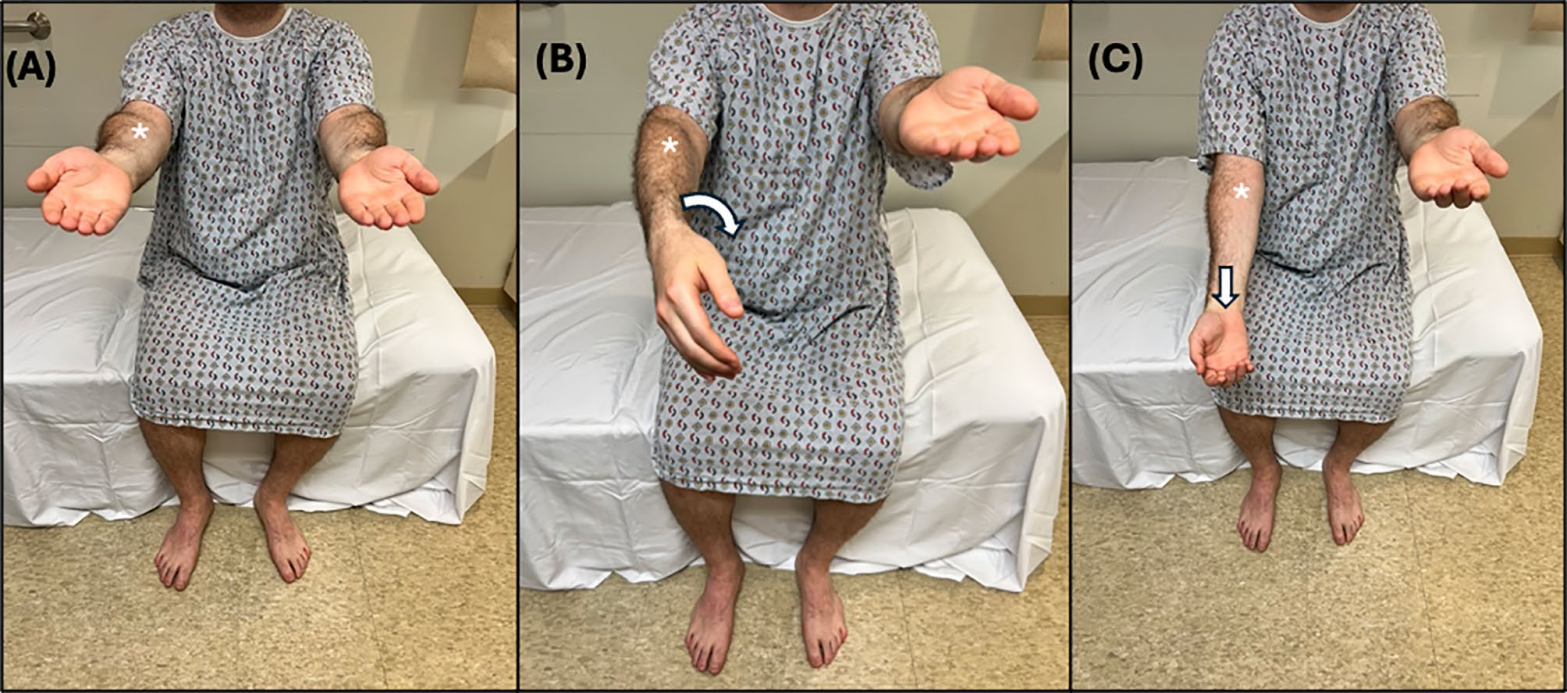
**A** Initial UE positioning. **B** Shows both drift and pronation of the affected UE (*), signifying a negative test. **C** Shows drift without forearm pronation of the affected UE (*), signifying a positive test

The patient should be seated, ensuring their upper extremity (UE) free of any obstructing instruments, such as blood pressure cuffs or finger pulse-oximeters. Both UEs should be outstretched forward and parallel to the ground, with palms flat and fully supinated and fingers adducted (Fig. 5). The examiner then instructs the patient to close their eyes for 10 s while holding their UE in that position. The examiner observes closely for any pronation in the affected UE.

### Interpretation and limitations

The sign is considered positive if drift is present in the absence of pronation in that UE. Drift without pronation was validated in a study examining 26 patients with UE FLW and 28 controls with ONW [34]. In this unblinded study, all patients with FLW exhibited drift without pronation, though it was also observed in 2 patients with ONW, resulting in a sensitivity of 100% and specificity of 93%. It is worth noting that this study excluded patients with complete paralysis, as they displayed absent drift. Drift without pronation is best used to assess patients with mild to moderate UE weakness given the need to first assume a position held against gravity. Also, many patients have difficulty holding their UEs up in full supination for 10 s as baseline, which can potentially affect the accuracy of the examination results.

### Paradoxical wrist flexion

Paradoxical wrist flexion, a relatively recently validated sign of UE FLW, is founded on the principle that muscles can exert the most force when maximally shortened [30]. For example, biceps brachii is able to produce less force when activated with the elbow extended compared to when the elbow is bent at 90 degrees, and it can produce the greatest force when the elbow is fully flexed. This test uses this principle to detect inconsistencies in the patient’s exam. Specifically, the strength of wrist flexion should consistently be stronger when the wrist is in the flexed position compared to when it is in the extended or neutral positions.

#### Technique

The patient should be seated with UEs flexed at 90 degrees at the elbows. From this position, wrist strength is tested in two separate positions.

##### Test 1

**Fig. 6:**
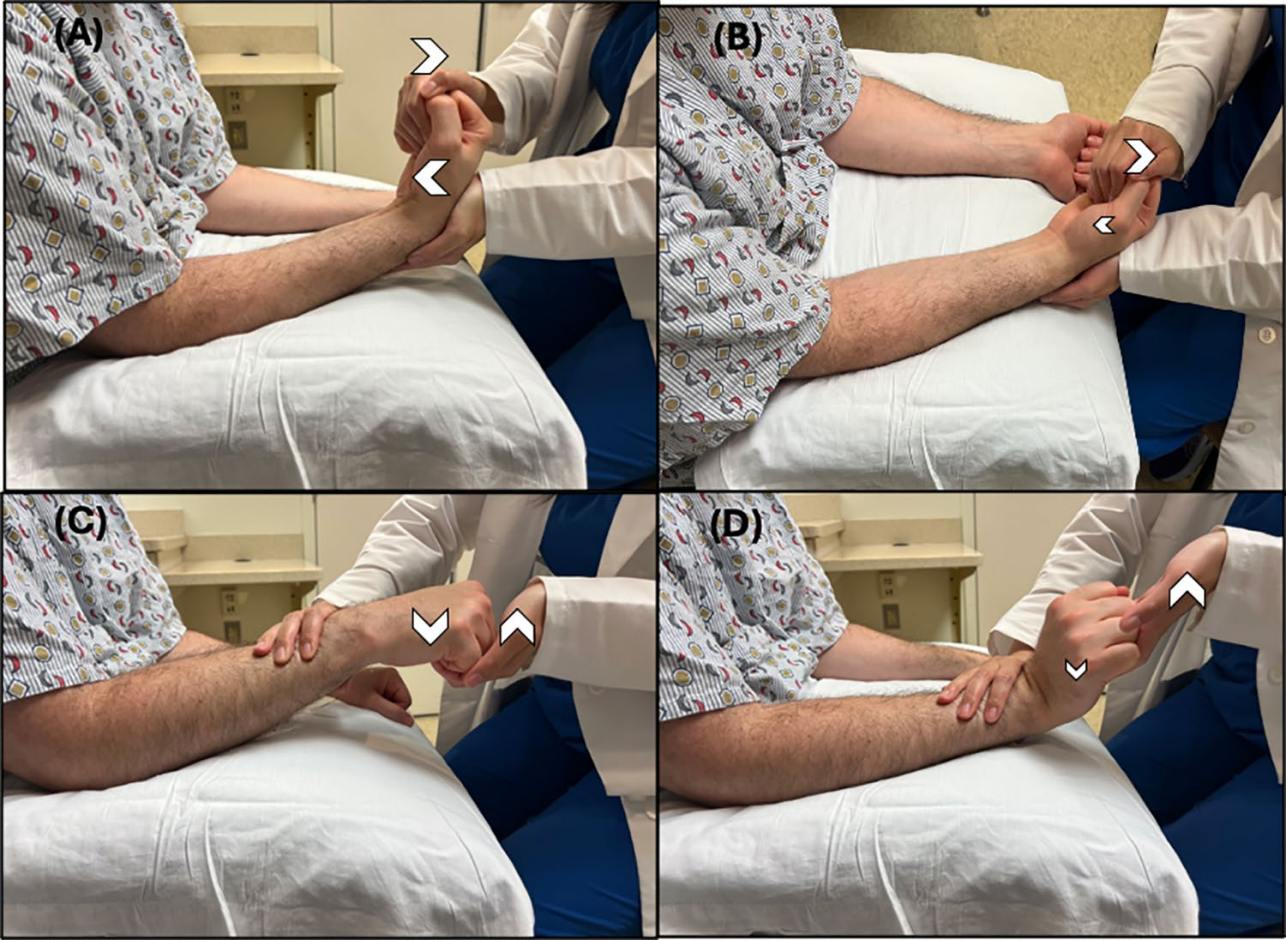
**A** Test 1: With the wrist supinated and flexed, the patient is asked to hold the wrist in flexion while the examiner provides a resistive force. **B** Shows a situation in which the examiner’s resistance could overcome the flexion strength of the patient. **C** Test 2: With the wrist pronated and wrist in neutral position, the patient is asked to hold the wrist in place while the examiner provides resistive force. **D** Show a situation in which the examiner could overcome the strength of the patient

The examiner positions the affected UE so that the palm is supinated, with fingers adducted and held in a fist position (Fig. 6). Then the wrist is placed in a fully flexed position so that the patient’s fist is facing upward. The patient is then asked to provide maximum resistance against the examiner as the examiner attempts to extend the patient’s wrist to the neutral position. Specific attention is paid to the strength of wrist flexion.

##### Test 2

The examiner passively places the patient’s wrist so that the palm is pronated and the fingers are adducted and held in a fist position. Next, the wrist is positioned in a neutral position, in-plane with the forearm. The patient is then asked to provide maximum resistance against the examiner as they attempt to extend the patient’s wrist upward. Specific attention is paid to the strength of wrist flexion.

### Interpretation and limitations

Paradoxical wrist flexion is considered present if more wrist flexion force is generated by the patient when the wrist is pronated and in the neutral position than when it is in the supinated and flexed position. Thus, FLW is expected if the patient produces more force performed test 2 than performing test 1.

The study demonstrated 24 patients with FLW, that the presence of paradoxical wrist flexion has a sensitivity of 67% and a specificity of 100% for distinguishing FLW from ONW. Evaluation for this sign is only appropriate in patients with mild to moderate weakness. Pain in the wrist or prior wrist surgery may also confound the test result.

## Elbow flex-ex sign

The elbow Flex-ex sign is another relatively newly validated positive sign of FLW [31]. This technique aims to distinguish UE FLW from ONW based on the same synergistic movement principle used in LE examination techniques such as Hoover’s sign, specifically the principle of complementary opposition.

### Technique

This test can be performed in either the standing or seated position, with UEs placed out in front. The examiner will position the patient’s elbows flexed at 30 degrees (so that the total angle from the patient’s shoulder to hand is 150 degrees). The examiner, positioned in front of the patient, then places both of his/her hands around both of the patient’s wrists.

#### Baseline strength assessment

Complete manual muscle testing is performed to establish a strength baseline, with specific attention paid to elbow flexion and extension.

##### Test 1

**Fig. 7:**
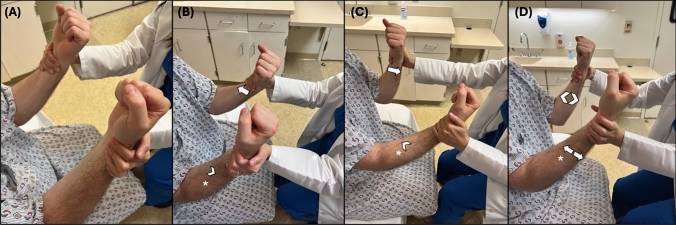
**A** Baseline assessment. **B** Test 1, part 1: The patient is instructed to flex the unaffected UE at the elbow against resistance while the examiner pays specific attention to any extension force provided by the affected UE (*), and how it compares to that found during (**A**). **C** Test 2, part 2: The patient is instructed to extend the unaffected UE at the elbow against resistance while the examiner pays specific attention to any flexion force provided by the affected UE (*), and how it compares to that found during (**A**). **D** Test 2, parts 1 and 2. The patient is asked to flex the affected UE (*) against resistance, while paying specific attention to any extension force of the unaffected UE. This is repeated with the patient being instructed to extend the affected UE and the examiner noting any flexion force provided by the unaffected UE. Here the arrow signifies the action and direction of the instructed action, and the arrowhead signifies the action and direction being assessed

The patient is instructed to flex the unaffected UE at the elbow (Fig. 7). With the examiner holding both the patient’s UE in place at the wrist, the examiner takes specific notice of any oppositional movement in the contralateral affected limb. For example, if the unaffected limb provides flexion force against the examiner, an extension force may be present in the contralateral affected limb. Next, the patient is asked to extend the unaffected UE at the elbow, and the examiner again pays specific attention to any flexion force applied by the contralateral affected limb.

##### Test 2

The patient is instructed to flex the affected UE at the elbow. With the examiner holding both the patient’s UEs in place at the wrist, the examiner takes specific notice of any oppositional movement in the contralateral unaffected limb. The patient is then asked to extend the affected UE at the elbow, and the examiner again pays specific attention to any flexion force applied by the contralateral unaffected limb.

### Interpretation and limitations

Test 1 is considered positive if during flexion or extension of the unaffected UE, a greater amount of oppositional extension or flexion force is felt in the affected UE compared to baseline.

Test 2 is considered positive if, during flexion of the affected UE, poor extension force is felt in the unaffected UE compared to baseline.

During the validation study, test 2 was determined to be neither sensitive nor specific. Thus, this test should be considered positive, and thus elbow flex-ex sign present if test 1 is positive. The presence of the elbow flex-ex sign is an indicator of likely FLW.

The study validating this technique examined 54 participants, 10 with FLW, 21 with ONW, and 23 with no weakness [31]. This study showed 100% sensitivity and specificity in differentiating FLW from ONW using the elbow flex-ex sign when the first part of the exam (Test 1) was positive. Examiners were not, however, blinded to the etiology of patients’ weakness prior to examination.

## Discussion

The interpretation of exam findings consistent with FLW should be made in the context of a full neurological examination and thorough patient history. A single positive sign indicative of FLW should not be interpreted as independently confirmatory. Though not confirmed, the reliability of test results may be greater if physical examination exploits multiple known physiological properties, such as complementary opposition and cross-body synkinesis. Additionally, verifying finding that utilize one property using multiple exam maneuvers may be helpful. For example, one can test for Hoover’s sign as well as abductor sign, which share the principle of complementary opposition.

Also, there are also other validated tests such as the sternocleidomastoid test or platysma sign that do not directly test the weakness of the limb in question, but rather assess for FLW elsewhere [25, 42]. These signs have been shown to co-occur in patients with FLW, with high specificity (90–100%) and moderate sensitivity (63%). The decision regarding which exam maneuvers to use is highly situation-dependent, though studies suggest that when able, testing for multiple signs likely increases both yield and positive predictive value over testing for only one [24].

Furthermore, though only validated in either the upper or lower extremity, the principles used in some techniques may be reasonably modified for examining other regions. For example, the principle behind the ability of a muscle to exert the most force when maximally shortened, as tested in the paradoxical wrist flexion test, may also be extrapolated for use in lower extremities, (i.e., examining footdrop). However, interpreting exam findings from techniques performed outside of their validated use, for example interpreting findings from a paradoxical wrist flexion or paradoxical elbow flexion modified for examination of the ankle or knee, respectively, should be done with caution as these techniques have not been validated for these body regions.

By using the exact techniques of the validated studies above, as well as utilizing multiple examination techniques with various underlying principles, it is likely that examiners can provide patients with a more accurate weakness assessment. With studies showing that patients with FLW often present with multiple signs of FLW, the utilization of multiple motor, gait, and sensory exam technique is likely to help to improve diagnostic certainty as well as minimize the risk of misdiagnosis [34]. Greater diagnostic confidence may in-tern expedite appropriate treatment, and reduce additional psycho-socio-economic costs the patient may incur due to delayed diagnosis, as well as medical waste for the care provider.

Lastly, it has been reported that a significant minority (~ 19%) of those with ONW also have at least one sign of FLW [43]. Comorbid anxiety and sensory deficits were found to be the most significant risk factors for patients with ONW and positive signs of FLW. Thus, it is important to complete a thorough neurological exam on those with positive signs of FLW, as many are likely to have additional ONW findings. Additionally, there are limitations to the generalizability of the findings in these studies. It should be considered that though several of the discussed techniques report a sensitivity or specificity of 100%, these statistics should be interpreted with caution as some studies have a low number of participants. Also, several studies included examinations performed or interpreted by an unblinded researcher. This unblinded assessment has the potential to bias the interpretation of the exam findings, and future exam validation studies should consider blinded exam interpretation to minimize potential bias.

## Conclusion

There are several validated examination techniques that can be used to distinguish FLW from ONW. Here, we have reviewed not only the exact validated exam maneuvers, but also provided guidance on how clinicians should interpret their exam findings given these results. Though many techniques have been reliably validated, some remain unclear in terms of the exact technique that should be used. By examining weakness using the techniques described in these validation studies, as well as utilizing multiple underlying principles of motor reflex and strength, clinicians may be able to reduce diagnostic uncertainty, and, in turn, improve the care of their patients.
